# Histomorphometric analysis of the skin of women during the reproductive period

**DOI:** 10.6061/clinics/2018/e387

**Published:** 2018-10-25

**Authors:** Heraldo Carlos Borges Inforzato, Adriana Aparecida Ferraz Carbonel, Ricardo Santos Simões, Gisela Rodrigues da Silva Sasso, Patricia Daniele Azevedo Lima, José Maria Soares-Júnior, Lydia Masako Ferreira, Manuel de Jesus Simões

**Affiliations:** I Divisao de Cirurgia Plastica, Universidade Federal de Sao Paulo (EPM/UNIFESP), Sao Paulo, SP, BR; II Departamento de Morfologia e Genetica, Universidade Federal de Sao Paulo (EPM/UNIFESP), Sao Paulo, SP, BR; III Departamento de Ginecologia e Obstetricia, Faculdade de Medicina FMUSP, Universidade de Sao Paulo, Sao Paulo, SP, BR; IV Department of Cellular and Molecular Medicine, University of Ottawa, Ottawa, Canada

**Keywords:** Skin, Epidermis, Dermis, Collagen

## Abstract

**OBJECTIVES:**

The aim of this study was to evaluate the histomorphometry of the skin of women during the reproductive period according to the Fitzpatrick classification.

**METHODS:**

Thirty women aged 30 to 45 years were included in this study. We studied the surgical sites of extracted nevi. The material was processed for routine histology and then stained with haematoxylin and eosin as well as Picrosirius red. Four-micrometre histological sections were analysed according the Fitzpatrick criteria (skin pigmentation). The skin thickness and collagen concentration were determined for the reticular dermal skin. The data were statistically analysed with ANOVA.

**RESULTS:**

Fitzpatrick skin types I and II were thicker than the other skin types.

**CONCLUSIONS:**

Our data suggest that white skin may be less thick than dark skin.

## INTRODUCTION

Since the appearance of the first human ancestor, our skin has played a significant role as an organ responsible for protection, vitamin D3 synthesis, thermoregulation and protection against folic acid lyses 1-4.

Edwards and Duntley 5 were the first to quantify human skin pigmentation. They used a spectrophotometer to measure the visible spectrum of their own pigmentation. However, Schulze 6, in 1956, was the first to consider human skin according its reaction to UV rays.

Fitzpatrick 7 established the classification criteria related to reactions to sun rays based on a specific need to classify white-skinned people undergoing phototherapy.

Currently, the most frequently applied skin classification method is the one established by Fitzpatrick 8. He presented a classification based on six different skin types 9,10.

Regarding morphological aspects, there are rare studies in the literature that mention the different types of skin and their histomorphometric characteristics. A majority of studies suggest that there is no difference in skin thickness between black and white people 4,11-13.

Shuster et al. 14 suggest that women's skin thickness is continuous until they reach 50 years of age. This characteristic is not observed in male skin, even though women's skin contains less collagen. Oriá et al. 15 also suggested that skin thickness decreases with human age because of hormone changes.

Montagna and Carlisle 16 did not observe a difference in facial skin thickness between black and white women.

Based on these data, this study conducted a morphological and morphometric study (thickness and collagen concentration) of female skin during the reproductive period based on the Fitzpatrick classification method.

## MATERIALS AND METHODS

The procedure applied in this study was first submitted to and approved by the UNIFESP/EPM Ethics Committee, and it was carried out after the women's assent was obtained. Skin samples were collected from women at the Santa Rita Clinical Centre in Guarujá/São Paulo and analysed.

Skin samples were taken from 30 eumenorrheic women aged 20 to 45 years. Their skin had previously been classified by two observers according to the parameters established by Fitzpatrick 10 (Table 1). Some exclusion factors were defined, including skin and collagen diseases, the use of hormone-based medications, the absence of menstruation and a lack of exposure to the sun exposure in the last 30 days or more.

Women classified with skin types V and VI and presenting with a dysplastic nevus were not identified in the sample and were not studied. This pathology is very rare in people with darker skin because they have greater natural protection from melanin 17.

After the local application of lidocaine (2%) and adrenalin (1:200.000), skin fragments were extracted from the free surgical margins of a dysplasia nevus in the dorsal region. The fragments were immersed in 10% formaldehyde and phosphate buffer for 12 hours and then processed for paraffin inclusion. The fractions (3 mm) were submitted to staining with haematoxylin and eosin (H.E.) and Picrosirius red (Sirius Red F3BA - Sigma-Aldrich Corp., St. Louis, MO).

### Morphometric analysis

The thickness of the H.E.-stained fractions was measured on images taken with image-capture equipment. The images were obtained with a high-resolution camera (AxioCam – Carl Zeiss^®^) attached to a Zeiss microscope (Carl Zeiss^®^) that transmitted these images to a Pentium 4 computer with 502 megabytes of RAM memory working on the Windows XP Professional^®^ platform.AxioVision Rel 4.2 (Carl Zeiss^®^) software was employed for the skin measures. It was standardized with a millimetric scale plate (Carl Zeiss^®^) for the several objectives used (4, 10, 40X). This procedure was performed for 20 epidermal measures from each woman limited by the under-face of the basal layer (the portion in contact with the basal membrane) and the outermost face of the stratum granulosum (the layer in which the nucleus is under the lucidum stratum) in different sections of the same plate. The section chosen was the outermost part of the dermal papilla.

The same procedure was applied to determine dermal thickness. The upper limit was the under-portion of the under-face of basal layer, and the under limit was the subcutaneous adipose tissue. The section chosen for these measures was the under-section of the epidermal papilla.

To determine the collagen concentration, the Picrosirius red-stained fragments were used. Ten images from the reticular dermis were taken from the middle dermis portion of each woman. The image capture system was the same as that described above. These images were analysed using Image-Pro-Plus^®^ software version 4.5.

### Statistical analyses

One-way ANOVA was used to analyse data analysis of skin thickness and collagen concentration data. The dermis and epidermis thickness in the same group was statistically compared using the Bartlett test, while the Bonferroni test was used for comparisons between the different types of skin. The same scheme was followed for the collagen concentration analyses.

## RESULTS

### Morphological results

Among fragments of the same skin type, there were no significant morphological differences. However, some differences between the epidermis and dermis thickness were observed. Thus, well-defined epidermis and dermis limits were observed in all skin types.

**Figure 1:**
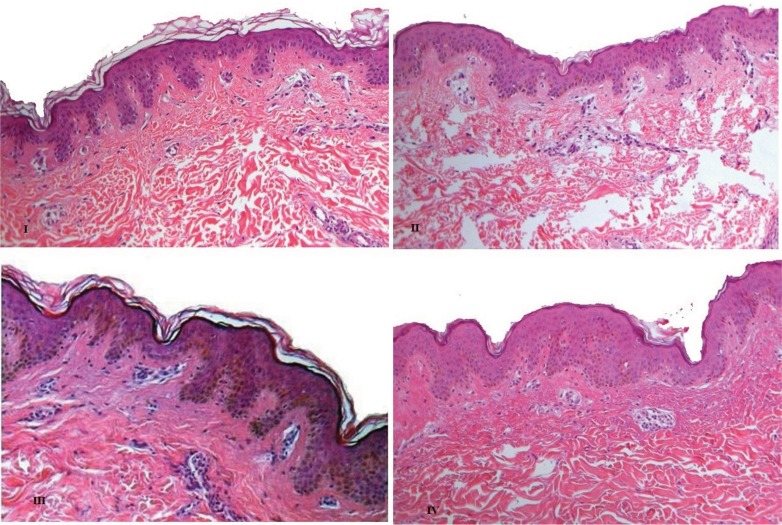
Photomicrographs of Fitzpatrick skin types I, II, III and IV in women.

Skin types I and II presented a thin epidermis and well-defined epidermal papilla compared with the other types. These types had a lower quantity of granulose and spiny keratinocytes compared to the other types (Figure 1). In the type III and IV epidermis, melanin was clearly present in the basal and spiny keratinocyte cytoplasm layer (Figure 1).

The presence of loose connective tissue between the dermal papilla of all studied types was noted. Numerous collagen fibres were regularly distributed under the dermal extension (reticular region).

In skin types I and II, numerous light gaps were identified between the collagen fibre bundles. In skin types III and IV, these bundles were thicker.

### Morphometric results

Analyses of epidermis (*p*=0.18) and dermis (*p*=0.32) thickness and collagen concentration (*p*=0.458) showed no discrepancies among the skin types studied. The Bartlett test was applied to elucidate the homoscedasticity principle (Table 2).

## DISCUSSION

The human species has undergone considerable miscegenation that has resulted in the appearance of different skin shades, from white to black. Considering that skin shade can be associated with various pathological statuses, it is necessary to understand the architecture of the skin and establish a relationship between skin architecture and skin colour.

Various studies have attempted to classify the skin, but the most frequently referenced is that of Fitzpatrick 7, who presented his first classification in 1975. He observed adverse effects when chemo- and phototherapy were used to treat psoriasis in people with different skin shades. In 1988, Fitzpatrick 8 presented a new classification that was adopted in this and other studies 18,19.

Women's skin was analysed in this study as women represent the patient population most likely to seek skin treatments and plastic surgery. Some limits were established to avoid deep hormonal interferences: the study participants were limited to women aged between 20 and 45 years with regular menstrual cycles.

Changes in skin characteristics during ageing are frequently determined by environmental and extrinsic factors, such as UV radiation. Therefore, the skin analyses performed in this study used samples collected from the dorsal zone of eumenorrheic women. This zone was chosen because it is not exposed to the environment full-time and because it presents a well-defined *lucidum stratum* that can make the epidermis measurements easier.

The 30 patients were well distributed among skin types I to IV. There were no patients with skin types V and VI because the skin fragments were taken from the free surgical margins of excised dysplastic nevi, and this type of pathology is rare among people with those skin types.

Histological differences between the black and white skin have been reported in other articles. A few of these make some reference to the intermediary pigmentation. Lu et al. 20 were the first authors to make an analogy between the Fitzpatrick classification method and the melanin granule distribution in keratinocytes of different skin types. This analogy seems to be logical and suitable for clinical practice.

Several studies of melanocytes have suggested that melanosomes differ between black and white people. In 1991, Montagna and Carlisle 16 demonstrated that the epidermis of black skin has thicker collagen fibres, larger fibroblasts and larger melanosomes. It should be noted that this study included samples from white, light brown and black skin. The use of three skin types reinforces the need for parameters for histological studies of the different skin types. Instead limited studies to three skin types, this new parameter must reflect a larger diversity that is complementary to anthropological studies.

Significant differences in the thickness of the skin and its layers (dermis and epidermis) were found between the different skin types classified according to Fitzpatrick. Skin type I was the thinnest, and skin type IV was the thickest. The majority of authors of previous studies had studied the skin from corpses, and they focused on neither age nor gender. In addition, there was no standardization of the region from which the skin samples were collected. It is important to clarify that the thickness and viscoelastic properties of the skin depend on the quantity of the material quantity in the dermis and its structural organization 11,12.

To determine the collagen concentration in the dermis, Picrosirius staining based, which is based on Sirius Red staining, was applied. As this stain is strongly acidic, it reacts with the amine groups of the lysine molecules in the collagen structure. Microscopic analysis revealed that the collagen from skin types I and II is similar in morphology and concentration. The same is true for skin types III and IV; however, no significant differences (*p*=0.458) were observed between types I and II and types III and IV. It is important to reiterate that this method evaluates the collagen concentration of a predetermined fraction of the study samples.

The data obtained could be useful to surgical and clinical practices because it indicates the patient groups who have more or less protection against the environment because their epidermis is thinner or thicker. This could be one more factor that explains why white skin is more inclined toward skin cancer, which confirms the information found in the medical literature. Considering skin healing, darker skin tends to respond faster to healing after surgical incisions, which reflects the higher keloid incidence in this group. However, the dermis of people with skin types I and II may have less tensile strength than the dermis of people with skin types III and IV.

These data can be applied in surgical and clinical areas in relation to protection factors, healing and laser and cosmetic treatments for the different skin types.

Regarding the clinical application of our findings, we believe that there are biological differences in skin. Thicker skin is more resistant to stress and tends to wrinkle less because it has a higher collagen fibre content. Furthermore, there may be differences in the absorption of topical pharmacological substances and a great tendency toward collagen and fibroblast proliferation, which may help during the cicatricial process or explain the high number of keloids among patients with these skin types. Such characteristics are due to a high quantity of stem cells, fibroblasts, collagen fibres, and other elements of the extracellular matrix, such as structural proteins and glycosaminoglycans which contribute to different inflammatory processes 21.

Our data analysing the skin of Brazilian women considered Brazil as a mixed country with great variation in ethnic groups. According to the Fitzpatrick classification method, lighter skin is thinner. However, there was no difference in the collagen concentration in the dermis according to skin type.

## AUTHOR CONTRIBUTIONS

Inforzato HC and Carbonel AA were responsible for general supervision, data acquisition and writing and revising the manuscript. Simões RS, Azevedo Lima PD and Sasso GR were responsible for discussion, writing and final preparation of the manuscript. Soares-Júnior JM, Ferreira LM and Simões MJ were responsible for the final preparation of the manuscript.

## Figures and Tables

**Table 1:** Skin types according to Fitzpatrick 8 skin type classification in relation to the first solar exposure of the summer.

Skin	Pigmentation	Skin reaction to the first summer exposure
I	White	Burns and never tans
II	White	In general, burns but can tan with difficulty
III	White	Sometimes burns, can get moderately tanned
IV	Light brown	Sporadically burns but tans easily
V	Light brown	Tans very easily and never gets burned
VI	Black	Tans very easily

**Table 2:** Morphometric analysis of Fitzpatrick skin types I, II, III and IV 8.

Skin types according to the Fitzpatrick classification				
	Type I (n=6)	Type II (n=8)	Type III (n=10)	Type IV (n=6)
Skin thickness (mm)	2.99±0.22	3.49±0.37	3.91±0.21	4.30±0.82
Epidermis thickness (µm)	40.39±1.05	44.10±1.07	50.49±2.11	58.72±1.54
Dermis thickness (mm)	2.97±0.21	3.45±0.35	3.86±0.19	4.24±0.71
Collagen in the reticular dermis (%)	61.83±5.58	67.66±2.89	70.38±2.45	64.44±2.74

## References

[1] Quevedo WC, Fitzpatrick TB, Pathak MA, Jimbow K (1975). Role of light in human skin color viariation. Am J Phys Anthropol.

[2] Jablonski NG, Chaplin G (2000). The evolution of human skin coloration. J Hum Evol.

[3] Linton R (2000). O homem. Tradução Lavínia Vilela. 12a Ed.

[4] Langaney A, Cloítes J, Guilaine J, Simonnet D (2002). A mais bela história do homem. Tradução Maria Helena Kühner. 1a Ed.

[5] Edwards EA, Duntley SQ (1939). The pigment and color of human skin. Am J Anat.

[6] Einige SR (1956). Versuche und Bemerkungen zum Problem der Handelsueblichen Lichtschutzmittel. Parf u Kosm.

[7] Fitzpatrick TB (1975). Soleil et peau. J Med Esthet.

[8] Fitzpatrick TB (1988). The validity and practicality of sun-reactive skin types I through VI. Arch Dermatol.

[9] Jimbow K, Quevedo WC, Fitzpatrick TB, Szabo G (1976). Some aspects of melanin biology: 1950-1975. J Invest Dermatol.

[10] Fitzpatrick TB (1986). Ultraviolet-induced pigmentary changes: benefits and hazards. Curr Probl Dermatol.

[11] Olson RL, Gaylor J, Everett MA (1973). Skin color, melanin, and erythema. Arch Dermatol.

[12] Weigand DA, Haygood C, Gaylor JR (1974). Cell layers and density of Negro and Caucasian stratum corneum. J Invest Dermatol.

[13] Rubegni P, Cevenini G, Barbini P, Flori ML, Fimiani M, Andreassi L (1999). Quantitative characterization and study of the relationship between constitutive-facultative skin color and phototype in Caucasians. Photochem Photobiol.

[14] Shuster S, Black MM, McVitie E (1975). The influence of age and sex on skin thickness, skin collagen and density. Br J Dermatol.

[15] Oriá RB, Ferreira FV, Santana EN, Fernandes MR, Brito GA (2003). Estudo das altera&ccedil;&otilde;es relacionadas com a idade na pele humana, utilizando m&eacute;todos de histo-morfometria e autofluoresc&ecirc;ncia. An Bras Dermatol.

[16] Montagna W, Carlisle K (1991). The architecture of black and white facial skin. J Am Acad Dermatol.

[17] Joel L. Spitz (2005). Genodermatoses. A Clinical Guide to Genetic Skin Disorders.

[18] Sadick NS, Smoller B (2009). A study examining the safety and efficacy of a fractional laser in the treatment of photodamage on the hands. J Cosmet Laser Ther.

[19] Sachdeva S (2009). Fitzpatrick skin typing: applications in dermatology. Indian J Dermatol Venereol Leprol.

[20] Lu H, Edwards C, Gaskell S, Pearse A, Marks R (1996). Melanin content and distribution in the surface corneocyte with skin phototypes. Br J Dermatol.

[21] Weigand DA, Haygood C, Gaylor JR (1974). Cell layers and density of Negro and Caucasian stratum corneum. J Invest Dermatol.

